# Re-emergence of arbovirus diseases in the State of Rio de Janeiro, Brazil: The role of simultaneous viral circulation between 2014 and 2019

**DOI:** 10.1016/j.onehlt.2022.100427

**Published:** 2022-08-10

**Authors:** Olivia M. Man, Trevon L. Fuller, Joelle I. Rosser, Karin Nielsen-Saines

**Affiliations:** a David Geffen School of Medicine, UCLA, 10833 Le Conte Ave, Los Angeles, CA 90095, USA; b Institute of the Environment and Sustainability, UCLA, 619 Charles E Young Drive East, La Kretz Hall, Suite 300, Box 951496, Los Angeles, CA 90095, USA; c Stanford University School of Medicine, Stanford, 291 Campus Drive, Stanford, CA 94305, USA

**Keywords:** Brazil, Dengue, Zika, Chikungunya, Arboviruses, Environment

## Abstract

The burden of arbovirus diseases in Brazil has increased within the past decade due to the emergence of chikungunya and Zika and endemic circulation of all four dengue serotypes. Changes in temperature and rainfall patterns may alter conditions to favor vector-host transmission and allow for cyclic re-emergence of disease. We sought to determine the impact of climate conditions on arbovirus co-circulation in Rio de Janeiro, Brazil. We assessed the spatial and temporal distributions of chikungunya, dengue, and Zika cases from Brazil's national notifiable disease information system (SINAN) and created autoregressive integrated moving average models (ARIMA) to predict arbovirus incidence accounting for the lagged effect of temperature and rainfall. Each year, we estimate that the combined arboviruses were associated with an average of 8429 to 10,047 lost Disability-Adjusted Life Years (DALYs). After controlling for temperature and precipitation, our model predicted a three cycle pattern where large arbovirus outbreaks appear to be primed by a smaller scale surge and followed by a lull of cases. These dynamic arbovirus patterns in Rio de Janeiro support a mechanism of susceptibility enhancement until the theoretical threshold of population immunity allows for temporary cross protection among certain arboviruses. This suspected synergy presents a major public health challenge due to overlapping locations and seasonality of arbovirus diseases, which may perpetuate disease burden and overwhelm the health system.

## Highlights

• Simultaneous circulation of dengue, chikungunya, and Zika in Brazil may strain the health system.
• Changing rainfall and temperature may allow for cyclic re-emergence of arboviruses.
• Predictive models with lagged climate variables can estimate patterns of disease.

## 1 Introduction

The burden of arbovirus related diseases is elevated and increasing in the Americas, infecting millions of people each year [[1], [2], [3]]. Several arbovirus infections can have long-term consequences such as hemorrhage and shock secondary to dengue infection, chronic post-infectious arthritis after chikungunya, and microcephaly and developmental delay in infants of mothers infected with Zika virus [[4], [5], [6]]. Brazil is thought to contribute to a large proportion of the global arbovirus disease burden due to the optimal ecologic conditions, dense urban areas, large land mass, and a prevalent mosquito vector population [[7], [8], [9]]. Prior studies have suggested that intermittent water supply and water storage, as lower income households are less likely to be connected to water infrastructure, creates additional breading sites for the *Aedes aegypti* [7]. Within the past decade, Brazil has experienced simultaneous circulation of all four dengue serotypes at endemic levels, the emergence of both chikungunya and Zika as well as an unusually large outbreak of yellow fever virus [[10], [11], [12]]. Large infectious disease outbreaks, such as SARS-CoV-2 or arboviruses, can overwhelm the health care system and exacerbate morbidity and mortality [9,13].

Cyclic re-emergence and transmission of arboviruses has been attributed to the dynamic interplay between humans, the environment, and *A. aegypti* mosquitoes [14,15]. Arboviruses are thought to have emerged from non-human primates, which do not usually show signs of clinical disease but can maintain viral titers high enough to sustain transmission though arboreal mosquitoes [16]. This sylvatic transmission cycle can spill over and infect humans by ways of increased human contact with forested environments (e.g., deforestation, urbanization) or when infected arboreal mosquitoes migrate into human habitats [16]. After arboviruses are transmitted to humans, infections can spread rapidly through the population by urban mosquitoes, such as the *A. aegypti*. Variations in climate patterns may shift the spread of arbovirus infections to new locations and create conditions that favor vector to human transmission [8,17]. High levels of population immunity following endemic outbreaks can give an opportunity for competing arboviruses to predominate [7].

Mosquito vectors have been previously shown to have strong seasonal patterns with incidence of arbovirus infections in humans related to season, temperatures, humidity, climate zone, deforestation, and rain level [18]. However, while there is general agreement that certain environmental variables are integrally related to arbovirus transmission, researchers have found variations in the effect of these environmental variables on transmission by location, year, and virus [12,19,20]. For example, two meta-analyses noted significant heterogeneity in the relationship between temperature and dengue incidence [21,22] and mechanistic modeling studies estimated wide temperature ranges for dengue, chikungunya, and zika transmission [23,24].

To date, the majority of studies have focused on specific arbovirus transmission [25,26] and few have examined the practical implications of simultaneous outbreaks on the health system. In areas where multiple arboviruses co-circulate, it can be difficult to clinically differentiate between infections due to overlapping symptom presentations. Given the limited availability and cost of laboratory tests and the lack of disease-specific medical treatments, laboratory confirmation is often not performed. Accurate arbovirus surveillance is also limited by the fact that arbovirus serologic testing by enzyme linked immunosorbent assays (ELISAs) is susceptible to cross reactivity between different arboviruses (e.g., between dengue and Zika) [26].

We evaluated the impact and dynamics of the co-circulation of dengue, chikungunya, and Zika viruses in the state of Rio de Janeiro, Brazil. First, we calculated disability adjusted life years (DALYs) to estimate the burden of arbovirus diseases in the state of Rio de Janeiro. We then examined then environmental conditions, co-circulation, and epidemic outbreak cycles of dengue, chikungunya, and Zika over 6 years throughout this state. Finally, we created autoregressive integrated moving average models (ARIMA) to predict arbovirus incidence accounting for the lagged effect of temperature and rainfall.

## 2 Methods

### 2.1 Study site and population

**Fig. 1:**
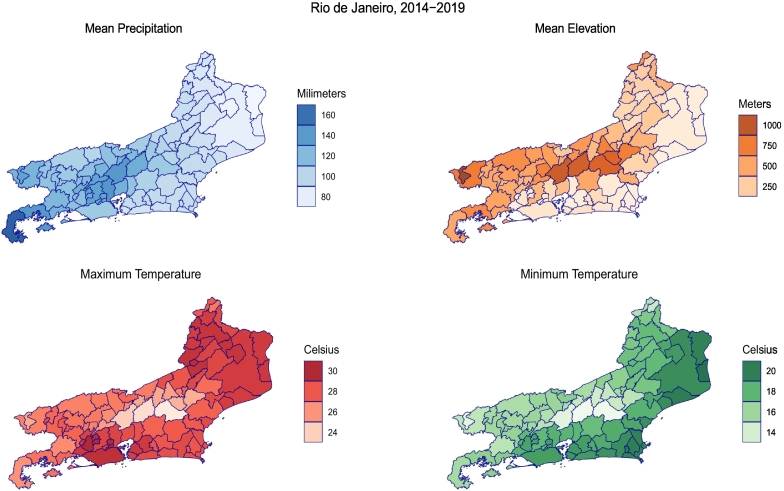
Average municipality climate conditions (2014–2018) in the state of Rio de Janeiro, Brazil. The average rainfall in the state of Rio de Janeiro decreased in 2017 and steadily rose during the rainy months of 2018 and 2019. On average, temperature patterns appeared to be relatively consistent between 2014 and 2018. (For interpretation of the references to colour in this figure legend, the reader is referred to the web version of this article.)

Rio de Janeiro was an epicenter of the 2016 Zika epidemic and is the second most densely populated state in Brazil, with a population density of approximately 383 people per square kilometer [27]. The region is located along the southeastern coast of Brazil and is comprised of 92 administrative municipalities. Rio de Janeiro has a tropical climate with higher temperatures along the lower lying costal municipalities and lower temperatures in the central municipalities with higher elevations (Fig. 1). Rainfall is concentrated in the hotter summer months and usually peaks in January.

### 2.2 Data sources

Weekly laboratory confirmed cases of chikungunya, dengue, and Zika cases were obtained from Brazil's national notifiable disease information system (SINAN) from 2014 to 2019. SINAN is a countrywide passive surveillance system that receives information from hospitals and outpatient clinics through a standardized form [28]. In Brazil, it is mandatory to report all suspected cases of chikungunya, dengue, and Zika to the health department. We obtained monthly information on precipitation, temperature, and elevation from WorldClim with a 2.5-min resolution [29].

### 2.3 Case definitions

Laboratory confirmed cases of chikungunya, dengue, and Zika were determined using guidelines from the Ministry of Health. Briefly, suspected cases were reported to SINAN based on key clinical symptoms, duration of symptoms, and recent travel to areas with known infections. Cases were confirmed using serology and molecular laboratory techniques. Due to the similar clinical presentation of chikungunya, dengue, and Zika, we only selected laboratory confirmed cases for our analysis to minimize misclassification. Finally, because 2016 coincided with the emergence and rapid expansion of the previously undetected Zika virus and the known ELISA cross reactivity between flaviviruses, we reclassified all 2016 laboratory confirmed dengue cases as Zika, in accordance with surveillance studies of the Zika epidemic in Rio de Janeiro [30]. Yellow Fever was not assessed as it has been previously analyzed elsewhere and was a sylvatic epidemic propagated by non-Aedes species mosquitoes, which likely have different responses to environmental stressors [12].

### 2.4 DALY calculations

We used templates from the World Health Organization (WHO) to calculate the yearly average disability adjusted life years (DALYs) lost from the three arboviruses [31]. Briefly, DALYs are a proxy to estimate burden of disease, combining years of life lost due to premature mortality (YLLs) and years of life lost due to living with disability (YLDs). We first used countrywide estimates from the Pan American Health Organization to estimate the population proportions for each sex and age category and the state specific census to obtain the age and sex adjusted population distributions. We used disability weight categories estimated from the Global Burden of Disease study [32]. For chikungunya, a meta-analysis found that approximately 40% of cases did not fully recover from an acute infection [4]. Therefore, we estimated that 60% of infections would have a mild acute episode, corresponding to a disability weight of 0.006, and 40% of infections would have severe long-term effects, with a disability weight of 0.219. For dengue, we adapted the methods from Stanaway et al. and considered 94.5% of infections to have an acute moderate episode, with a disability weight of 0.051, and 5.5% of infections to have an acute severe episode, with a disability weight of 0.133 [3]. For Zika, we assumed that the majority of cases would have a mild symptomatic presentation and a disability weight of 0.006. Based on results from a meta-analysis, we assigned 2.3% of Zika pregnancies with a disability weight of 0.179, corresponding to fetal alcohol syndrome, to account for children born with microcephaly [33].

### 2.5 Environmental conditions and spatial assessment

We investigated the environmental risk factors and simultaneous circulation of each arbovirus across the state of Rio de Janeiro from 2014 through 2019. We created summary maps of mean precipitation, mean elevation, maximum temperature, and minimum temperature for the entire time period to identify average climate conditions for each municipality. Next, we created yearly incidence maps for each arbovirus to assess disease spread over time. We plotted monthly time series graphs at the state level to assess the timing of environmental changes and infections.

### 2.6 Environmental model

We summed laboratory confirmed cases of dengue, chikungunya, and Zika to calculate the total number of arbovirus cases in Rio de Janeiro per month from January 2014 to December 2019 and fit a predictive Autoregressive Integrated Moving-Average (ARIMA) time series model [34] to the combined arbovirus cases. In an ARIMA model, the events of interest in a given month are modeled as a linear combination of the number of events in previous months. To determine whether the time series of arbovirus cases was suitable to analyze with an ARIMA model, we used an autocorrelation test for white noise processes to evaluate the null hypothesis that the time series of arbovirus cases was a purely random process with no significant information. After carrying out this diagnostic test, we constructed an ARIMA model in which the arbovirus cases in a given month are modeled as a linear combination of the number of cases in past months along with current and past values of temperature and precipitation. The analysis utilized maximum daily temperature, which was highly correlated with other measures of temperature. We assessed the fit of the parameters of the ARIMA model to the observed arbovirus data using a *t*-test. We assessed the goodness of fit of the model to the arboviruses cases using Q-Q plots, autocorrelation function bar charts, and via a t-test of the model coefficients.

## 3 Results

### 3.1 Burden of arbovirus disease

A total of 425,220 suspected cases of chikungunya, dengue, and Zika, with 0.15% mortality, were reported in the state of Rio de Janeiro, Brazil from 2014 to 2019 (Table 1). Of the suspected cases, only 42,636 (30%) chikungunya, 56,861 (41%) dengue, and 133,413 (24%) Zika cases were confirmed. We calculated that combined between 8429 and 10,211 DALYs were lost annually for the three arboviruses evaluated. The majority of DALYs were lost due to dengue, followed by chikungunya, and lastly Zika.

**Table 1:** Total cases and disability adjusted life years (DALYs) of Chikungunya, Dengue, and Zika in Rio de Janeiro Brazil, 2014–2019.

	**Chikungunya**	**Dengue**	**Zika**^^^	**Total**
Clinical Cases	142,120	140,151	142,949	425,220
Laboratory Confirmed Cases	42,636	56,861	33,916	133,413
Average yearly DALYs Clinical	3389	4799	1859^#^	10,047
Average Yearly DALYs Lab	2636	4270	1523^#^	8429

^: Zika cases are likely underestimated due to clinical case definitions requiring a maculopapular rash or misclassification from cross reactivity with dengue for laboratory serologies.

#: While Zika DALYs include children born with microcephaly, they do not account for the lost DALYs from caretakers.

### 3.2 Temporal distribution of arboviruses and environmental conditions

**Fig. 2:**
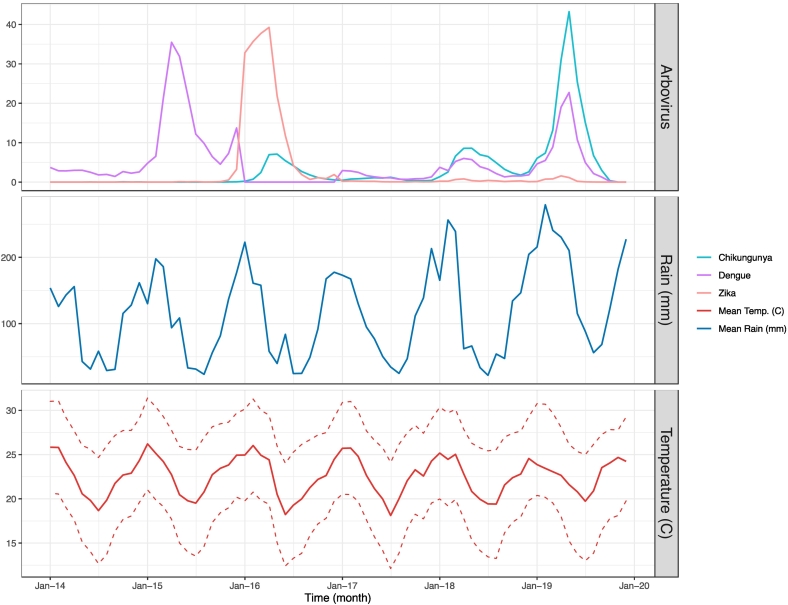
Monthly time series (2014–2019) of Chikungunya, Dengue, and Zika incidences per 100,000, average temperature (Celsius) and average precipitation (mm) in the state of Rio de Janeiro. Temperature is bounded to illustrate mean maximum and mean minimum temperatures for the region. Climate date from 2014 to 2018 was obtained from WorldClim using a 2.5 resolution [28]. Climate data from 2019 was obtained from the European Centre for Medium-Range Weather Forecasts Reanalysis 5 [37].

Overall, *A. aegypti-*transmitted arbovirus cases were high in Rio de Janeiro in 2015–2016, plummeted in 2017–2018, before resurging again in 2019 (Fig. 2). Dengue was the primary circulating arbovirus, with the highest incidence based on laboratory confirmed cases compared to chikungunya and Zika incidence, from 2015 to 2016. Zika cases peaked during 2016, when it became the primary circulating virus. Chikungunya incidence gradually increased over time and became the primary circulating arbovirus in 2018–2019. The majority of arbovirus outbreaks occurred after large rainfall events in warmer months.

### 3.3 Spatial distribution of arboviruses

**Fig. 3:**
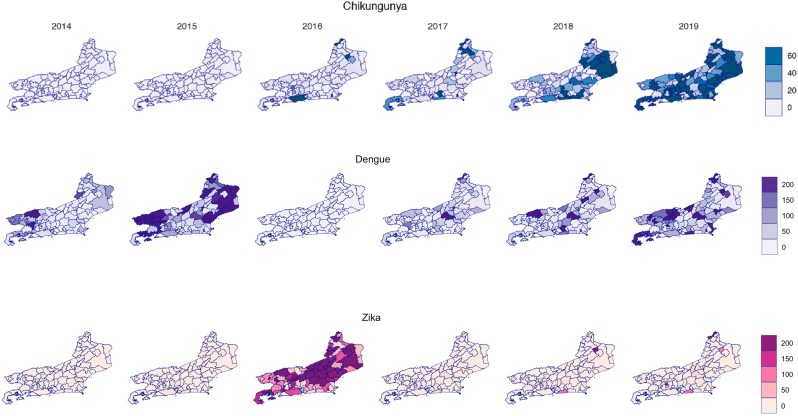
Spatial distribution of yearly arbovirus incidence per 100,000 in Rio de Janeiro from 2014 to 2019. Dengue virus infections peaked in early 2015, declined in 2016 when the Zika virus epidemic peaked and resurged in 2019. An initial increase in Chikungunya infections was observed in 2017, with progression across the state in 2018 and dissemination across most state municipalities in 2019.

In the state of Rio de Janeiro, municipalities with higher levels of dengue were located in the eastern and western parts of the state from 2014 to 2015 (Fig. 3). From 2017 to 2019, we observed increasing dengue incidence sporadically throughout the state. In contrast, evidence of larger chikungunya outbreaks throughout municipalities in the north and the southern coast started to emerge in 2016. Chikungunya appeared to spread to neighboring municipalities in 2017, before expanding at high levels throughout the state in 2018–2019. Finally, Zika incidence was highest in municipalities along the southern coast in 2015 before spreading throughout the state in 2016. Incidence of Zika receded briefly in 2017 and appeared to be increasing in more central parts of the state in 2018–2019.

### 3.4 Environmental model

**Fig. 4:**
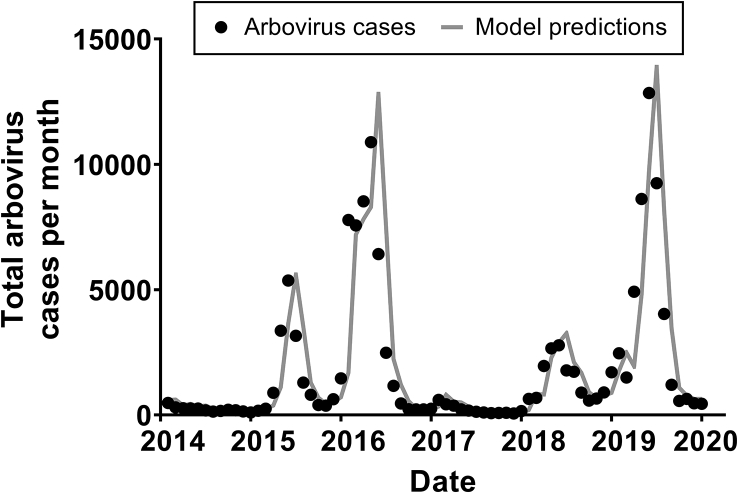
Total reported arbovirus cases per month in the state of Rio de Janeiro, 2014 to 2019 [27], and predicted arbovirus cases were based on a time series model incorporating temperature and precipitation.

From 2014 to 2019, the average maximum daily temperature was 36.1 °C. In each municipality, temperature and precipitation were highest from the months of November to March, which coincides with the austral summer. Arbovirus cases were also highest during this same period of the year. Diagnostic tests indicated that the time series of arbovirus cases was suitable to analyze using an ARIMA model. In particular, the null hypothesis that the time series was a purely random process was rejected (chi-squared = 128.44, *p* < 0.0001). Furthermore, diagnostics indicated that an ARIMA model constructed using precipitation and temperature to predict the timing of arbovirus cases provided a good fit to the observed arbovirus cases based on the Q-Q plot, autocorrelation function, and the *t*-test of model coefficients, which was highly significant (*t* = 17.87, p < 0.0001, Fig. 4). After controlling for temperature and precipitation, our model predicted smaller numbers of combined arbovirus cases in 2015 and 2018, followed by larger outbreaks in 2016 and 2019.

## 4 Discussion

We explored the burden of arbovirus diseases and how seasonal variations impact vector-host transmission dynamics across multiple outbreak cycles in Rio de Janeiro, Brazil. Using a national hospital-based surveillance system, we estimated a large burden of arbovirus diseases, approximated by an average of 8429 to 10,047 lost DALYs each year in the state. Peaks in arbovirus incidence appeared to occur after large rainfall events. Additionally, our environmental model was able to predict the timing of arbovirus cases based on monthly precipitation and temperature.

Tropical climates, such as the one in Rio de Janeiro state may have the potential to sustain longer periods of arbovirus transmission due to optimal vector conditions, such as temperature and precipitation. We found that, across all municipalities, arbovirus cases coincided with the austral summer, which had the highest yearly temperatures and rainfall. Previous studies have found the optimal transmission of chikungunya, dengue, and Zika occurs from 18 to 34 degrees, with maximum transmission between 26 and 29 degrees Celsius [23]. While Rio de Janeiro may have the optimal temperature for arbovirus transmission year-round, previous studies have found that both large and fine scale variations in temperature may impact transmission. For example, authors have noted that for every 1-degree Celsius increase in monthly temperature, the risk of dengue in humans increases by 1.95 [35]. Other research suggests that the temperature at the onset of the outbreak may be associated with the magnitude of cases [24]. On the other hand, seasonal peaks have previously been associated with fine scale variations in temperature, which may change mosquito behavior and facilitate potential outbreak oscillations beyond what a monthly time scale would suggest [23]. We observed arbovirus incidence peaks after months with a high average rainfall, which is consistent with previous literature [7]. This corresponds to the late rainy season, suggesting that there may be a lagged relationship between rainfall and incidence. While some authors have suggested that higher precipitation is associated with increased vector populations, extreme rainfall events may wash out containers and negatively impact the mosquito population [35]. Finally, while it appears that previous outbreaks have corresponded to hot rainy seasons, more recent outbreaks have shown sustained transmission into the dry season (Fig. 2, Fig. 4). This has been attributed to water storage practices in areas with inconsistent municipal water supply [7]. Changing climate patterns may shift the burden of arbovirus disease, increasing transmission to previously naïve populations or creating optimal conditions to sustain transmission for longer periods of time. While there has been limited evidence supporting current sylvatic transmission of dengue, chikungunya, and Zika in the Americas, risk of spillback transmission to further infect zoonotic populations may perpetuate future disease cycles [16].

Simultaneous circulation of arboviruses has been found in many municipalities at a yearly time scale (Fig. 3), which may create several diagnostic and treatment challenges for the health system. The spatiotemporal overlap of infections may suggest possible interactions between different arboviruses. Our environmental model shows large peaks in total arbovirus cases in 2016 and 2019, which were both preceded by a smaller number of cases the prior year (2015 and 2018) (Fig. 4). In other words, after adjusting for temperature and precipitation, we have observed a pattern of smaller scale outbreaks that appear to be priming a larger outbreak in the subsequent year. Interestingly, the year following the large outbreaks appear to have a nadir of arbovirus cases. Previous literature has proposed both temporary cross protection and susceptibility enhancement mechanisms. Experts have hypothesized that the lull in 2017 of *A. aegypti-*transmitted arbovirus cases can be attributed to residual immunity from elevated levels of both dengue and Zika in the prior year [36]. The decline in cases could also be due to more intense vector control efforts following the Zika epidemic. On the other hand, others have noted a suspected synergy between different arboviruses [37], which has allowed for simultaneous outbreaks in the area and may explain the smaller number of cases priming large, peaked outbreaks in our study. It is possible that arboviruses exhibit a level of susceptibility enhancement, which peaks at a theoretical threshold of population immunity before temporary cross protection can be observed.

Yellow fever was not included in this analysis because recent transmission in Brazil has been attributed to non-Aedes vectors [12]. However, laboratory studies have found *A. aegypti* to be a competent vector, suggesting a theoretical risk of yellow fever entering an urban transmission cycle with spread through the *A. aegypti* [12]*.* If yellow fever dynamics shift to involve the *A. aegypti* and human infection, this can further complicate the interactions between the different arboviruses and alter patterns of transmission.

There are some limitations to our analysis. While our study was designed to capture large scale variations in temperature and precipitation at a monthly resolution, it does not account for the effect of small-scale climate variations (e.g., extreme rain events or daily temperature changes) on arbovirus incidence. However, it is unclear if the impact of fine scale climate changes will be adequately identified by a passive surveillance system. This method of case identification is likely an underestimate of the true values because it fails to identify the individuals who do not seek health care or individuals with minor symptoms. Thus, it is possible that the environmental drivers causing micro level changes in transmission will ultimately be attenuated by the nature of the data collection system. The passive nature of SINAN data may explain the low incidence of Zika cases as only 20% of cases are symptomatic. Moreover, individuals are only considered to be a suspected Zika case if they present with a maculopapular rash [28]. It is likely that the laboratory confirmed cases greatly underestimate true disease burden in Rio de Janeiro during this time period. Clinical case counts were 3.3, 2.5, and 4.2 times higher than laboratory confirmed cases for chikungunya, dengue, and Zika respectively. Thus, when considering the burden of arbovirus diseases on the population, we used clinically suspected and laboratory confirmed cases to calculate a range of average DALYs lost. In addition, during the Zika epidemic of 2015–2016, PCR testing was prioritized for pregnant women. Therefore, laboratory case confirmation of Zika virus infection in the overall population was minimal, particularly because serologic testing (e.g., ELISA) for Zika virus was not reliable due to cross-reactivity with dengue virus in a setting where the majority of the population has pre-existing dengue antibodies. To account for this, we reclassified all 2016 dengue cases as Zika in accordance previous studies [30].

## 5 Conclusion

This suspected synergy between arboviruses presents a major public health challenge due to overlapping locations and seasonality of disease. After adjusting for temperature and precipitation, smaller numbers of arbovirus cases appear to prime the population for a larger outbreak the subsequent year. Simultaneous presence of large outbreaks has the potential to overwhelm the health system and perpetuate disease burden. For this reason, it is critical to have efficient tools to predict patterns of disease based on specific environmental drivers.

## Funding sources

OM was supported by the Global Health Program at David Geffen UCLA School of Medicine. JIR was supported by NIH Training Grant 5T32AI052073–14. TF and KNS received support from AI140718 (NIH/NIAID).

None of the authors received financial or material support for the research and work in this manuscript. The funders had no role in study design, data collection and analysis, decision to publish, or preparation of the manuscript.

## CRediT authorship contribution statement

**Olivia M. Man:** Conceptualization, Methodology, Software, Formal analysis, Writing – original draft, Writing – review & editing, Visualization. **Trevon L. Fuller:** Conceptualization, Methodology, Formal analysis, Data curation, Resources, Writing – review & editing, Visualization, Supervision. **Joelle I. Rosser:** Conceptualization, Writing – review & editing. **Karin Nielsen-Saines:** Conceptualization, Methodology, Data curation, Resources, Writing – review & editing, Supervision.

## Declaration of Competing Interest

The authors declare that they have no known competing financial interests or personal relationships that could have appeared to influence the work reported in this paper.
